# The Nucleosome (Histone-DNA Complex) Is the TLR9-Specific Immunostimulatory Component of *Plasmodium falciparum* That Activates DCs

**DOI:** 10.1371/journal.pone.0020398

**Published:** 2011-06-08

**Authors:** Nagaraj M. Gowda, Xianzhu Wu, D. Channe Gowda

**Affiliations:** Department of Biochemistry and Molecular Biology, Pennsylvania State University College of Medicine, Hershey, Pennsylvania, United States of America; New York University School of Medicine, United States of America

## Abstract

The systemic clinical symptoms of *Plasmodium falciparum* infection such as fever and chills correspond to the proinflammatory cytokines produced in response to the parasite components released during the synchronized rupture of schizonts. We recently demonstrated that, among the schizont-released products, merozoites are the predominant components that activate dendritic cells (DCs) by TLR9-specific recognition to induce the maturation of cells and to produce proinflammatory cytokines. We also demonstrated that DNA is the active constituent and that formation of a DNA-protein complex is essential for the entry of parasite DNA into cells for recognition by TLR9. However, the nature of endogenous protein-DNA complex in the parasite is not known. In this study, we show that parasite nucleosome constitute the major protein-DNA complex involved in the activation of DCs by parasite nuclear material. The parasite components were fractionated into the nuclear and non-nuclear materials. The nuclear material was further fractionated into chromatin and the proteins loosely bound to chromatin. Polynucleosomes and oligonucleosomes were prepared from the chromatin. These were tested for their ability to activate DCs obtained by the FLT3 ligand differentiation of bone marrow cells from the wild type, and TLR2^−/−^, TLR9^−/−^ and MyD88^−/−^ mice. DCs stimulated with the nuclear material and polynucleosomes as well as mono- and oligonucleosomes efficiently induced the production of proinflammatory cytokines in a TLR9-dependent manner, demonstrating that nucleosomes (histone-DNA complex) represent the major TLR9-specific DC-immunostimulatory component of the malaria parasite nuclear material. Thus, our data provide a significant insight into the activation of DCs by malaria parasites and have important implications for malaria vaccine development.

## Introduction

Malaria is a major infectious disease in many countries of the tropical and subtropical regions of the world. Nearly half of the world population is at risk of infecting with malaria parasite [1]–[3]. Malaria caused by the parasite, *Plasmodium falciparum*, alone is responsible for nearly one million fatalities annually [4]. Efforts to produce an effective vaccine against malaria have not yet been successful due to gap in our knowledge on the molecular mechanisms involved in the development of protective immunity [5], [6].

Innate immunity characterized by the production of proinflammatory responses early during the infection plays an important role in controlling malaria and other pathogenic infections [6]–[10]. Innate immune responses also play a critical role in activating the adaptive immune system for the development of pathogen-specific protective immunity [11], [12]. Dendritic cells (DCs) are the specialized cells of the innate immune system that are central to both innate and adaptive arms of the immune systems [13], [14]. DCs are not only involved in the early sensing and controlling of invading pathogens by producing proinflammatory responses and potentiating these responses by activating NK cells to secrete IFN-γ, but also initiate and shape up cell-mediated and humoral immunity by inducing Th1/Th2 differentiation of T cells and antibody production by B cells [15]–[17]. Thus, DCs link the innate immune system to the adaptive immune system for shaping of an effective protective immunity against invading pathogens.

Central to the functions of DCs and other antigen presenting cells such as macrophages of the innate immune system is the expression of Toll-like receptors (TLRs), a family of evolutionarily conserved, signal transducing transmembrane proteins [18], [19]. TLRs are involved in the recognition of invading pathogens by interacting with conserved molecules called pathogen-associated molecular patterns (PAMPs) [20]–[22]. TLRs are expressed either on cell surfaces or on the luminal side of endosomal membranes and exhibit discrete specificity to PAMPs. TLR4 has been shown to recognize bacterial lipopolysaccharides, TLR9 has been shown to recognize the CpG ODN-containing motifs of bacterial DNA, and TLR2 recognizes diverse ligands such as lipoteichoic acid, lipoproteins, and GPIs. Upon interactions with PAMPs, TLRs transduce signals through their conserved cytoplasmic segments, activating MAPK and NF-κB cascades and inducing a wide range of immunological responses, including the production of cytokines and chemokines and the upregulation of cell adhesion molecules and costimulatory molecules [18]–[23]. Thus, recognition of PAMPs by TLRs allows the innate immune system to discriminate various pathogens and initiate pathogen-specific immune responses [20], [23].

Many of the early clinical manifestations of malaria infection, including fever and chills, correspond to the secretion of proinflammatory mediators by the cells of the innate immune system in response to parasite components released at high levels by the synchronous burst of schizont stage parasite-infected erythrocytes [24], [25]. Recently, we have demonstrated that, among various components that are released during the *P. falciparum* schizont burst, merozoites (MZs) are the major parasite components that trigger proinflammatory cytokine responses in DCs [26]. Using exogenous polycationic proteins and by enzymatic degradative studies, we have demonstrated that parasite DNA is the stimulatory constituent of MZs that activates cells by TLR9 recognition, and that complex formation with polycationic proteins is essential for the internalization of DNA by DCs. However, the nature of endogenous parasite proteins that complex with DNA, enabling it to enter DCs remains unknown. In the present study, using DCs obtained by the FLT3 ligand-induced differentiation of mouse bone marrow cells and mouse spleen DCs, we sought to investigate the nature of protein-DNA complex in *P. falciparum* that is responsible for the MZ-induced activation of DCs. The data show that parasite nucleosome is the predominant protein-DNA complex that is responsible for the TLR9-specific immunostimulatory activity of MZs.

## Materials and Methods

### Reagents

Cell and parasite culturing reagents, including sodium pyruvate, non-essential amino acids, 4-aminobenzoic acid, gentamicin and 2-mercaptoethanol and trypsin, *Staphylococcus aureus* micrococcal nuclease, DNase I, calf thymus histones, and Percoll® were purchased from Sigma-Aldrich (St. Louis, MO). Dulbecco's modification of eagle's medium (DMEM), roswell park memorial institute (RPMI) 1640 medium, and penicillin/streptomycin solution were from Invitrogen (Carlsbad, CA). Fetal bovine serum (FBS) was from Atlanta Biologicals (Lawrenceville, GA). Collagenase D was from Roche Applied Science (Mannheim, Germany), CpG ODN1826 was from Coley Pharmaceutical (Kanata, ON, Canada). Hoechst 33258 dye from Promega Corp. (Madison, WI). Chicken ovalbumin^323–339^ (OVA^323–339^) peptide was purchased from Peptides International, Inc. (Louisville, KY). Dr. Sergei Gregoryev, Department of Biochemistry and Molecular Biology, Penn State University College of Medicine, Hershey, Pennsylvania, provided human recombinant histones, H1, H2A, H2B, H3 and H4.

Human O-positive blood and O-positive plasma were from the Blood Bank, Hershey Medical Center Hospital, Hershey, PA. FMS-like tyrosine kinase 3 (FLT3) ligand expressing B16 cell line was generously provided by Dr. Glenn Dranoff, Harvard University Medical School [27]. Conditioned medium from FLT3-expressing B16 cells was prepared as reported previously [26] and used as a source of FLT3 ligand for the differentiation of mouse bone marrow cells to DCs (FL-DCs).

Duoset ELISA kits for measuring mouse TNF-α, IL-12p40 and IFN-γ were from R & D Systems (Minneapolis, MN). Anti-mouse CD11c antibody (clone N418) conjugated microbeads, mouse NK cell isolation kit, and anti-mouse CD90.2 antibody conjugated microbeads and magnetic columns for cell separation were from Miltenyi Biotec Inc. (Auburn, CA). Fluorescein isothiocyante (FITC)-conjugated antibodies against mouse CD3e (clone 145-2C11), CD11c (clone N418) and pan NK cells (clone DX5), phycoerythrin (PE)-conjugated anti-mouse NK1.1 antibody (clone PK136), PE-Cy5-conjugated antibodies against mouse CD80 (clone 16-10A1), hamster IgG (clone eBio299Arm) isotype control, CD86 (clone GL1) and rat IgG2aκ isotype control, and allophycocyanin (APC)-conjugated antibodies against mouse CD40 (clone 1C10) and rat IgG2aκ isotype control were from eBioscience (San Diego, CA). Rabbit anti-human H3 histone polyclonal antibodies was from Cell Signaling Technology (Beverly, MA).

### Ethics statement

The Institutional Animal Care and Use Committee of the Pennsylvania State University College of Medicine, Hershey, has reviewed and approved the protocols (No. 2001-146) for the use of animals in this study. The Institutional Review Board of the Pennsylvania State University College of Medicine, Hershey, has reviewed and approved the protocols (No. HY03-261EP) for the use of human O-positive blood and O-positive plasma received from the Blood Bank, Hershey Medical Center Hospital, Hershey, PA.

### Mice

The wild type (WT), OT-II transgenic mice, and TLR2, TLR9 and MyD88 knockout mice (all in C57BL/6J background) were housed in a pathogen-free environment. The animal care was in accordance with the institutional guidelines of the Pennsylvania State University College of Medicine.

### *P. falciparum* parasites culturing

*P. falciparum* parasites (3D7 strains) were cultured using O-positive human erythrocytes in RPMI 1640 medium containing 10% human O-positive plasma and 50 µg/ml gentamicin under 90% nitrogen, 5% oxygen and 5% carbon dioxide atmosphere as described [28]. The cultures were tested for mycoplasma contamination at 10–15 day intervals using MycoSensor PCR assay kit from Stratagene (La Jolla, CA).

### Fractionation of parasite components and isolation of polynucleosomes

The parasite nuclear material and polynucleosomes were prepared as reported previously [29], [30]. The infected red blood cells (IRBCs) at the late trophozoite and schizont stages were enriched by centrifugation of the suspensions of RBC and IRBC mixture from the culture harvests on 60% Percoll cushions at 1500 g for 15 min at 4°C. The enriched IRBC pellet was suspended in 20 volumes of 0.1% saponin in PBS, pH 7.2, vortexed, incubated on ice for 10 min, and centrifuged at 2,500 g at 4°C for 15 min. The pellet was resuspended in the above solution, vortexed, incubated for 10 min, and centrifuged. The parasite pellet thus obtained was washed 3 times with cold PBS, pH 7.2, and suspended in 10 volumes of lysis buffer (20 mM HEPES, pH 7.5, 10 mM KCl, 1 mM EDTA, 1 mM dithiothreitol, 1 mM phenylmethylsulfonyl fluoride (PMSF) and 1% Triton X-100) and incubated on ice for 5 min. The suspension was centrifuged at 5,000 g at 4°C for 10 min, and nuclear material was washed three times with the lysis buffer to remove membrane components. Subsequently, to extract loosely bound proteins, the nuclear material pellet was extracted with 5 volumes of buffer A (20 mM HEPES, pH 7.5, 0.2 mM EDTA, 0.3 M KCl, 3 mM MgCl_2_, 3 mM 2-mercaptoethanol, and 1 µM each of pepstatin, leupeptin, benzamidine, *N*-tosyl-*L*-phenylalanine chloromethyl ketone, *N*-ethylmaleimide and 0.4 mM PMSF), centrifuged and the supernatant containing proteins was collected. To the pellet (chromatin material) was added drop-wise an equal volume of buffer A containing 0.6 M KCl and 10% (v/v) glycerol with gentle stirring and incubated on ice for 10 min, homogenized gently using Dounce homogenizer, and centrifuged. The supernatant containing the extracted proteins was collected and the chromatin material was further extracted with 20 volumes of buffer A containing 0.4 M NaCl and 5% (v/v) glycerol, and centrifuged at 10,000 rpm at 4°C for 10 min. The chromatin pellet was suspended in 4 volumes of buffer A containing 0.65 M NaCl and 0.34 M sucrose and sonicated to obtain chromatin material as a soluble component (polynucleosomes). The process was repeated two times and the supernatants containing the soluble polynucleosomes were pooled. These polynucleosomes preparations contained, in addition to histones, low levels of bound non-histone proteins and were used in all studies described here.

**Figure 1:**
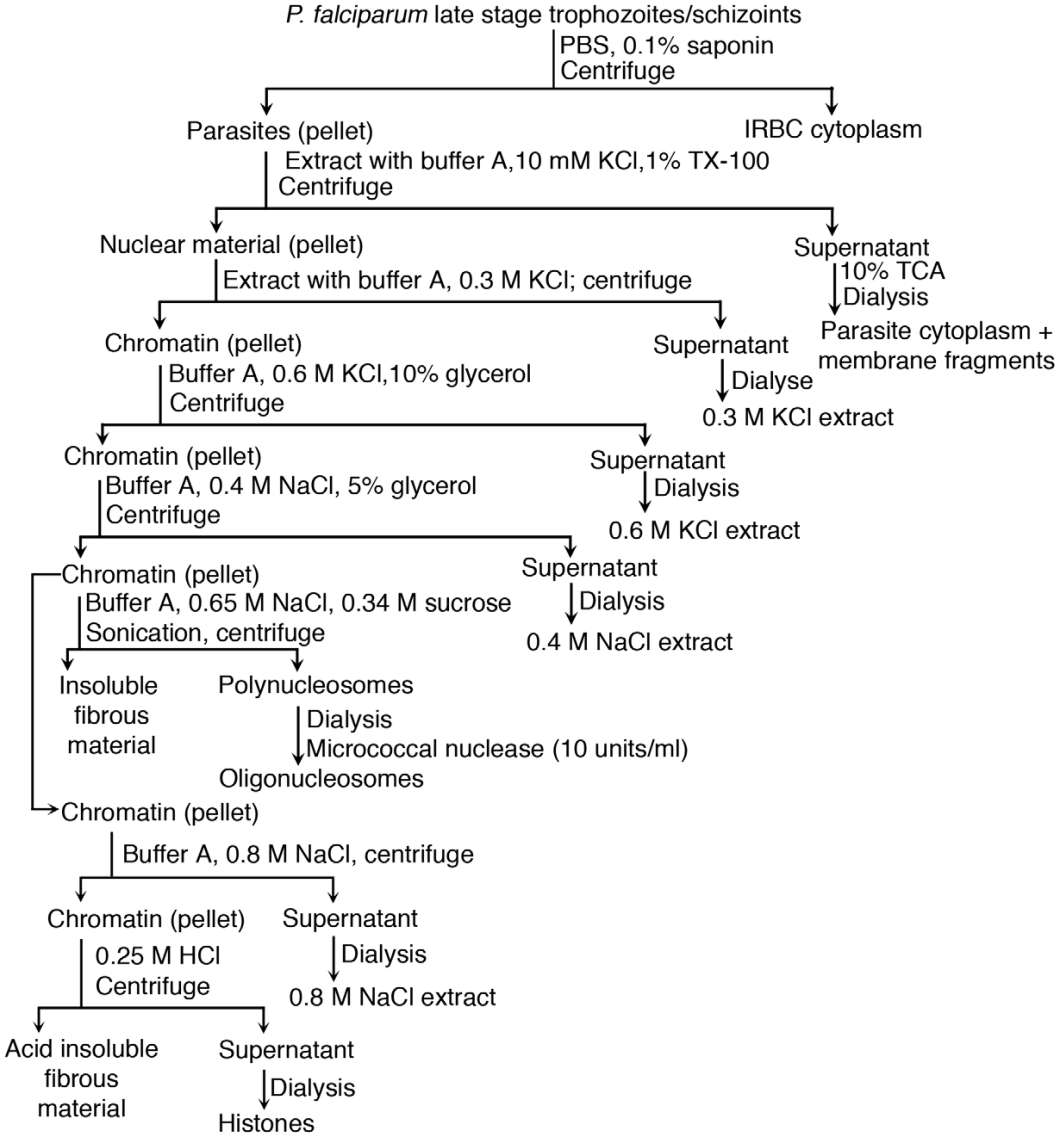
Fractionation of *P. falciparum* nuclear material and preparation of polynucleosomes and histones. The parasites released from the late trophozoite and schizoint stage-infected erythrocytes were lysed in buffer containing 1% Triton X-100. The insoluble nuclear material was pelleted, extracted with buffer having different salt concentrations to remove proteins loosely bound to the nuclear chromatin material. The chromatin material was sheared to yield polynucleosomes.

The various buffer/salt extracts (see above, and also see Figure 1) were dialyzed for 24 h with two changes of buffer A containing 0.1 M NaCl and protease inhibitors using 3.5-kDa molecular size cut off dialysis bags. Finally, the solutions were dialyzed overnight at 4°C against the same buffer but without protease inhibitors. The DNA concentration in the polynucleosome preparation was estimated by measuring absorption at 260 nm, and by spectrofluorimetry using Hoechst 33258 dye and measuring emission at 450 nm after excitation at 350 nm in ISS™ PC1 Photon Counting Spectrofluorimeter (ISS, Champaign, IL) [32]. The protein content was measured using a micro BCA protein estimation kit from Pierce (Thermo Scientific, Rockford, IL) [33]. Aliquots were analyzed by agarose gel electrophoresis, SDS-PAGE, and Western blotting.

### Preparation of parasite histones

To isolate parasite histones [31], the chromatin pellets prepared as above, were washed three times with 5 volumes of 0.8 M NaCl in buffer A and then extracted two times, each time, with 10 volumes of 0.25 M HCl, and allowed to stand on ice for 1 h with occasional mixing. The extracts were centrifuged separately in Eppendorf tubes at 12, 000 rpm for 5 min to remove insoluble debris. The 0.8 M NaCl and 0. 25 M HCl extracts (Figure 1) were dialyzed and DNA contents measured as described above for various parasite nuclear extracts. Aliquots of parasites extracts containing histones were analyzed by SDS-PAGE and then were pooled together for measuring stimulatory activity.

### Preparation of mono- and oligonucleosomes

The polynucleosomes (15 µg DNA content) in 200 µl of 20 mM HEPES, pH 7.5, containing 0.1 M NaCl, 3 mM CaCl_2_ and 0.5 µM PMSF was separately treated with different doses of micrococcal nuclease (0.5 to 50 units/ml) at 37°C for 5 min. The reaction was stopped by adding 0.1 volume of 0.5 M EDTA and chilling on ice, and 10 µl aliquots of the enzyme-treated samples were analyzed by electrophoresis using 2% agarose gel. The solutions were dialyzed against buffer A containing 0.1 M NaCl and were used for the stimulation of FL-DCs.

### Treatment of polynucleosomes with DNase and trypsin

The polynucleosomes (7.5 µg DNA content) in 100 µl of 20 mM HEPES, pH 7.5, containing 0.1 M NaCl and 0.5 µM PMSF was incubated with 100 µg/ml trypsin at 37°C for 30 min. The reaction was stopped by the addition of FBS to a final concentration of 20%. The non-treated polynucleosome solution incubated at 37°C for 30 min was used as a control stimulant. In a separate experiment, polynucleosome solution (100 µl, 7.5 µg DNA content) was incubated with DNase I (100 units/ml) at 37°C for 1 h. The enzyme was inactivated by the addition of 0.5 M EDTA to a final concentration of 2.5 mM and heating at 65°C for 10 min.

### Isolation of parasite genomic DNA

The IRBCs from parasite cultures harvested at the late trophozoite stage were lysed with 0.05% of saponin in cold PBS, pH 7.2. The released parasites were collected by centrifugation at 2,500 g, and washed two times with cold PBS, pH 7.2. Parasites were lysed with 10 mM Tris-HCl, pH 8.0, containing 20 mM EDTA, 0.5% SDS, and incubated with 25 µg/ml proteinase K at 56°C overnight. After diluting with an equal volume of water, the solution was extracted with phenol and chloroform and treated with RNase. The DNA was precipitated with 10 volumes of ethanol after the addition of 3 M NaOAc to a final concentration of 0.3 M. The DNA precipitated was washed with 70% ethanol, dried and dissolved in water. The DNA concentration was estimated by measuring the absorption at 260 nm and stored at −20°C until used.

### Isolation of mouse spleen cells

Mouse spleens were homogenized and centrifuged at 200 g for 10 min. The pellets were suspended in ammonium chloride containing RBC lysis solution, diluted with incomplete DMEM, and passed through 70-µm strainer to obtain a single cell suspension. NK cells were isolated by magnetic sorting using the mouse NK cell isolation kit. T cells from the spleens of OT-II mice were isolated using anti-mouse CD90.2 antibody-conjugated magnetic beads. For isolation of DCs, homogenized spleens were incubated with collagenase D (1 mg/ml in incomplete DMEM) at 37°C for 30 min, and a single cell suspension prepared as above. DCs were isolated by magnetic cell sorting using anti-mouse CD11c-conjugated microbeads; the purity of cells was ∼90%.

### Preparation of FLT3 ligand-differentiated DCs

Bone marrow cells from WT, TLR2^−/−^, TLR9^−/−^ and MyD88^−/−^ mice were cultured for 7 or 8 days in complete DMEM (DMEM containing 10% FBS, 1% penicillin-streptomycin, 1% non-essential amino acids, 1 mM sodium pyruvate, 50 µM 2-mercaptoethanol) supplemented with 15% of FLT3 ligand containing conditioned medium obtained by culturing B16 cells expressing retrovirus-coded FLT3 ligand [26], [34].

### Cell stimulation and cytokine analysis

DCs (1×10^5^/well) were seeded into 96-well flat-bottom plates and cultured in 200 µl complete medium, and stimulated with MZs, various parasite components or control ligands Pam_3_CSK_4_ (TLR2 ligand, 10 ng/ml), Poly I∶C (TLR3 ligand, 2 µg/ml), LPS (TLR4 ligand, 100 ng/ml) or CpG ODN (TLR9 ligand, 2 µg/ml) for 24 h. Unless otherwise indicated, the MZs and various parasite components were used at concentrations corresponding to 2.5 µg/ml DNA content. In experiments for results shown in Figures 5, 6, 8, 9, S1, S2, and S4, purified parasite genomic DNA (pDNA) was added at a concentration of 8 µg/ml. The culture supernatants were collected and cytokine levels measured by ELISA as reported previously [35]. In co-culturing experiments, DCs (10^5^ cells/well) and NK cells (0.5×10^5^/well) were cultured in 96-well U-bottom plates and stimulated with different doses of MZs or polynucleosomes in 200 µl of complete medium. After 36 h, the culture supernatants were harvested and analyzed for IFN-γ by ELISA. FL-DCs (1×10^5^/well in 96-well U-bottom plates) were activated with different doses of polynucleosomes for 6 h and then co-cultured with T cells (0.5×10^5^/well) purified from the spleens of OT-II naïve mice in the presence or absence of 2 µg/ml OVA^323–339^ peptide in 200 µl of complete medium. After 72 h, culture supernatants were harvested and assayed for IFN-γ by ELISA. In parallel, DCs, NK cells or T cells were cultured, stimulated, and culture supernatants used as controls for ELISA.

### Analysis of costimulatory molecules

For analysis of costimulatory molecules expression, FL-DCs (1×10^6^) were seeded in 24-well plates, cultured in 1 ml of complete DMEM and stimulated with *P. falciparum* nuclear material, polynucleosomes, MZs (each 2.5 µg/ml of DNA content) or CpG ODN (2 µg/ml). After 24 h, the cells were collected, stained with FITC-conjugated anti-mouse CD11c antibody, PE-Cy5-conjugated anti-mouse CD80 antibody and hamster IgG isotype control and APC conjugated anti-mouse CD40 antibody and rat IgG2aκ isotype control, PE-Cy5-conjugated anti-mouse CD86 antibody and rat IgG2aκ isotype control. After washing, the cells were analyzed by using Becton-Dickinson FACSCalibur flowcytometer and the results were analyzed with CellQuest software (BD Biosciences).

### Sucrose density gradient centrifugation

MZs (100 µl wet pellet), suspended in 1 ml of PBS, pH 7.2, were lysed by freezing and thawing, centrifuged and the supernatant was treated with 80% ammonium sulfate in buffer A. The precipitated proteins and protein-DNA-complexes were collected by centrifugation at 10,000 g for 20 min and dissolved in buffer A (1 ml), and dialyzed against buffer A containing 0.1 M NaCl and protease inhibitors. The suspension was loaded onto 20 ml of 10–20% sucrose gradient in buffer A and centrifuged in a Beckman Optima LE-80K ultracentrifuge using SW41Ti rotor at 85,000 g for 16 h. 0.5 ml fractions were collected from the top of the gradient using gradient collector. The fractions were dialyzed first against buffer A containing 0.1 M NaCl and protease inhibitors cocktail, and then against the same buffer without protease inhibitors. Optical density of fractions was measured at 260 nm and 280 nm. The concentrations of DNA and protein were estimated fluorimetrically using Hoechst 33258 dye [32] and by the micro BCA method [33], respectively. An aliquot of each fraction was analyzed by SDS-PAGE and by Western blotting. The fractions were used for stimulation of DCs.

### SDS-PAGE and western blot analysis

SDS-PAGE was performed under reducing conditions using 15% polyacrylamide gels and the gels were stained with Coomassie Brilliant Blue R250. For Western blot analysis, the protein bands in the polyacrylamide gels were transferred onto nitrocellulose membranes. The membranes were blocked with 5% (w/v) nonfat dry milk in PBS, pH 7.2, containing 0.1% Tween 20, at room temperature for 1 h, and then incubated with anti-H3 histone antibody in the above buffer containing 2.5% nonfat dry milk. After washing, the membranes were incubated with horseradish peroxidase-conjugated anti-rabbit secondary antibody, treated with chemiluminescent substrate (SuperSignal® West Pico Luminol from Thermo Scientific), and exposed to X-ray films.

### Statistical Analysis

The data were plotted as mean values ± SEM. Statistical analysis of the data was performed by Student's t tests and one-way analysis of variance followed by the Newman-Keuls test. GraphPad prism software version 3.0 was used for the analysis. *P* values<0.05 were considered statistically significant.

## Results

### The major DC immunostimulatory component of *P. falciparum* merozoites resides in the nucleus

**Figure 2:**
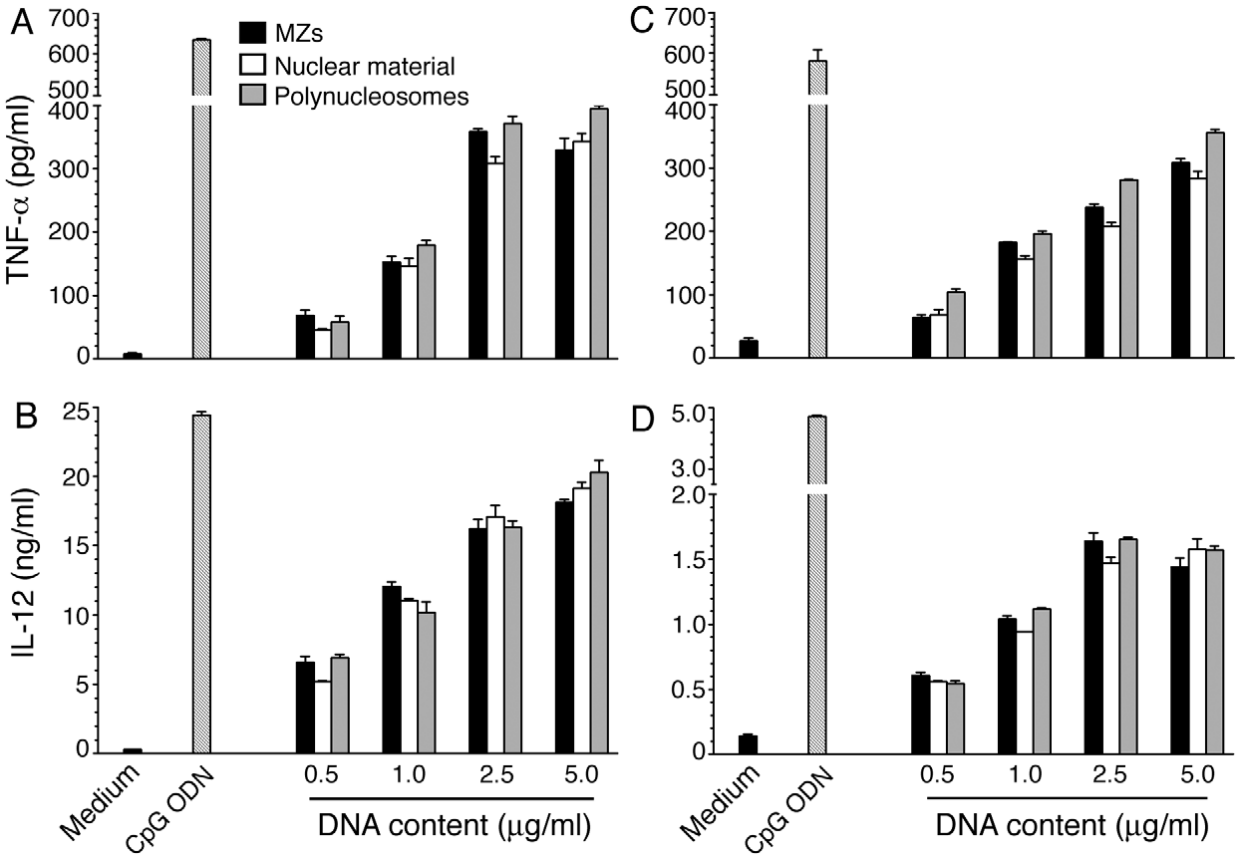
The nuclear material and polynucleosomes of *P. falciparum* efficiently activate DCs to produce inflammatory cytokines. FL-DCs (**panels A** and **B**) or spleen DCs (**panels C** and **D**) from WT mice were plated in 96-well plates and stimulated with the indicated doses (based on DNA contents) of nuclear material or polynucleosomes. FL-DCs similarly stimulated with merozoites (MZs, dose indicated by DNA content) or with a standard CpG ODN were analyzed as controls. The levels of TNF-α and IL-12 in the culture medium were measured by ELISA. Data are representatives of three independent experiments, each performed in duplicates. Error bar represents mean values ± SEM.

**Figure 3:**
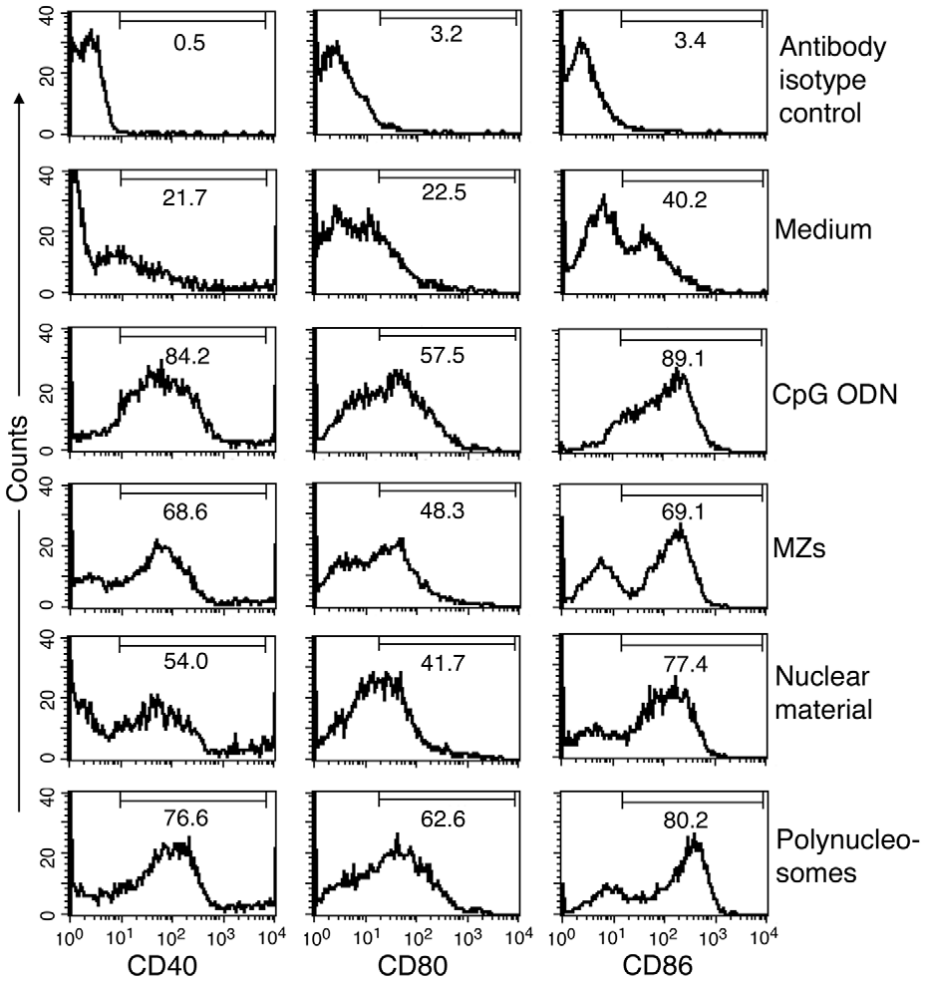
*P. falciparum* nuclear material and polynucleosomes induce the maturation of DCs. FL-DCs prepared from the bone marrow cells of WT mice were plated in 24-well plates and stimulated with merozoites (MZs), parasite nuclear material or polynucleosomes. DCs stimulated with MZs or CpG ODN was used as controls. The upregulated surface expression of costimulatory molecules, CD40, CD80 and CD86, were analyzed by flow cytometry. The percentages of DCs that are positive to each costimulatory molecule are indicated. Data are representatives of two independent experiments.

Recently, we showed that *P. falciparum* MZs potently activate DCs through TLR9 signaling pathway and that DNA is the predominant immunostimulatory molecule of MZs [26]. Although the MZs released during the rupture of the schizont stage malaria parasite infected erythrocytes are meant to invade erythrocytes, it is likely that only a fraction of the released MZs invade erythrocytes and the reminder is targeted by the innate immune system [36]–[39]. MZs are short lived and some of them likely undergo lysis *in vivo* similar to that has been observed *in vitro* [40]–[43], presumably releasing their cellular contents, including DNA. For parasite DNA to enter DCs and activate TLR9 signaling pathway, it must complex with polycationic proteins [26]. In the present study, in efforts to determine the nature of endogenous parasite protein-DNA complex, we first analyzed stimulatory activity of nuclear components of the parasite. We have previously showed that cytoplasmic and membrane components of IRBCs induced little or no inflammatory cytokine production, although they could activate DCs to upregulate the cell surface expression of co-stimulatory molecules to certain extent [26]. Therefore, in this study the IRBC cytoplasmic components of parasites were not analyzed. The parasites, released by treating the late trophozoite and schizont stage *P. falciparum*-infected erythrocytes with 0.1% saponin, were lysed with buffer containing 1% Triton X-100 to disrupt parasite vacuolar, plasma and nuclear membranes to yield nuclear material as a buffer-insoluble component (Figure 1) [29]. The nuclear material robustly activated both mouse FL-DCs and spleen DCs in a dose-dependent manner, inducing the production of TNF-α and IL-12 (Figure 2). On DNA equivalent basis, the purified *P. falciparum* MZs and the parasite nuclear material exhibited comparable levels of DC-stimulatory activity (Figure 2). The nuclear material also induced the maturation of DCs as revealed by the marked increase in the surface expression of costimulatory molecules, CD40, CD80 and CD86 (Figure 3). Collectively, these data strongly suggest that the nuclear material represent the major immunostimulatory component of *P. falciparum* MZs that efficiently activates mouse DCs.

### *P. falciparum* polynucleosomes (histone-DNA complex) efficiently activate DCs to induce proinflammatory cytokine responses

**Figure 4:**
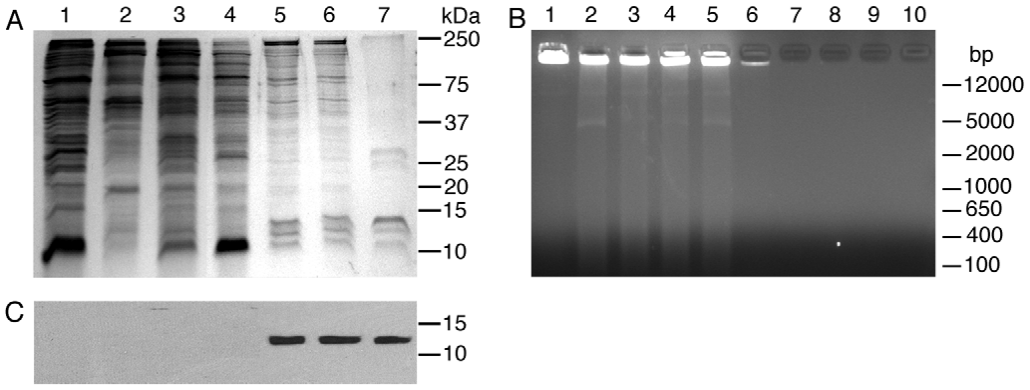
The analysis of the parasite nuclear material and extracts of nuclear material. **Panel A**: The parasite nuclear material, polynucleosomes and buffer/salt extracts of nuclear material were analyzed for proteins by SDS-PAGE (in each lane 15 µg of protein content/well). Lane 1, the supernatant of parasite lysate (see Figure 1 for materials in lanes 1–6); lane 2, 0.3 M KCl extract of the nuclear material; lane 3, 0.6 M KCl extract of the chromatin material; lane 4, 0.4 M NaCl extract of the chromatin material; lane 5, chromatin material after extraction of proteins with buffer A containing various salts; lane 6, polynucleosomes; lane 7, the mixture of standard recombinant histones, H1, H2A, H2B, H3 and H4. The mobility of molecular weight marker proteins is indicated to the right. **Panel B**: The parasite nuclear material and buffer/salt extracts of nuclear material were electrophoresed on 0.8% agarose gels and the DNA bands were visualized under UV light after ethidium bromide staining. Lane 1, parasite nuclear material before extraction of proteins that loosely bound to chromatin (see Figure 1 for materials in lanes 1–10); lane 2, chromatin material pellet after extraction with buffer/0.3 M KCl; lane 3, chromatin material after extraction with buffer/0.6 M KCl; lane 4, chromatin material after extraction with buffer/0.4 M NaCl; lane 5, soluble polynucleosomes; lane 6, insoluble fibrous material after obtaining polynucleosomes; lane 7, parasite cytoplasmic material plus membrane fragments; lane 8, 0.3 M KCl extract of nuclear material; lane 9, 0.6 M KCl extract; lane 10, 0.4 M NaCl extract. Lanes 1–5, materials having 0.5 µg DNA were analyzed. Lane 6, about 60-fold more material as compared to those in lanes 1–5 based on the total parasite amount used for fractionation. Lanes 7–10, materials equivalent to that in lanes 1–5 based on the total amount of parasite used for fractionation was loaded. The sizes of standard DNA makers are indicated to the right. **Panel C**: Western blotting of parasite components as shown in **panel A** was done using anti-H3 histone polyclonal antibodies. Lane descriptions are as outlined in **panel A**.

To characterize the nature of the parasite nuclear immunostimulatory component, the nuclear material, obtained as above, was extracted successively with buffers containing, (i) 0.3 M KCl, (ii) 0.6 M KCl and 10% glycerol, and (iii) 0.4 M NaCl and 5% glycerol, to remove proteins such as transcription factors that are loosely associated with chromatin (Figure 1), [30]. SDS-PAGE and agarose gel electrophoresis, and fluorimetric analysis using Hoechst 33258 dye indicated the presence of proteins but not DNA in the buffer/salt extracts of the nuclear material (Figures 4A, 4B, and data not shown). SDS-PAGE and agarose gel electrophoresis revealed that the chromatin material obtained after depleting most of the loosely bound proteins contained proteins and DNA (Figures 4A and 4B). Given that histones are the major proteins that avidly bind to DNA, we analyzed the chromatin material and the salt extracts by Western blotting using anti-H3 histone antibodies. The data indicated that the protein bands having electrophoretic mobility corresponding to the molecular mass of 10–15 kDa observed in chromatin material were histones (Figure 4A, lanes 5 and 6, and Figure 4C). Although some proteins corresponding to the molecular size of histones were observed in buffer/salt extracts of the nuclear material (Figure 4A), histones were not detectable in these fractions by Western blotting (Figure 4C, lanes 1–4). The buffer/salt extracts lacked DNA and showed no DC stimulatory activity and the activity was fully associated with the chromatin material (Figure S1). However, upon addition of parasite genomic DNA, the buffer/salt extracts efficiently stimulated DCs (Figure S1). Since histones strongly bind DNA, it is possible that the observed activity of these fractions is due to the presence of low levels of histones present in these fractions that were not detectable by Western blotting. Alternatively, the activity was due to some positively charged non-histone parasite proteins forming complex with DNA, facilitating its uptake by DCs.

In eukaryotic cells, the chromatin consists of mainly DNA and highly positively charged histones and is present as a condensed structure made up of tightly packed nucleosomes, the unit structures of condensed DNA. The nucleosomes are composed of 146 bp DNA winding around histone octomers and a ∼60-base pair long linker DNA to which H1 histone binds and helps in compact packing of nucleosomes [44]. The insoluble parasite chromatin material, obtained as above, was suspended in buffer containing 0.65 M NaCl and 0.34 M sucrose and sonicated to shear DNA. This process solubilized the majority of chromatin material, yielding soluble polynucleosomes containing high molecular weight DNA, histones, and appreciable levels of non-histone proteins (Figures 4A, lanes 5 and 6, and Figure 4B). FL-DCs were stimulated with polynucleosomes corresponding to 2.5 µg DNA content/ml, which is equivalent to 3×10^6^ IRBCs/ml of culture medium. The polynucleosomes robustly activated DCs in a dose-dependent manner, producing high levels of TNF-α and IL-12 (see Figure 2). Most of the DC-stimulatory activity of the parasite nuclear material was present in polynucleosomes and the insoluble fibrous material contained low levels of activity (data not shown); hence, the latter material was not further investigated. At various concentrations (based on DNA contents) tested, the levels of TNF-α and IL-12 produced by DCs stimulated with polynucleosomes were comparable to those of TNF-α and IL-12 secreted by DCs in response to the parasite nuclear material or whole MZs (see Figure 2). Further, the polynucleosomes efficiently induced the maturation of DCs as indicated by the marked upregulation of co-stimulatory molecules, CD40, CD80 and CD86, on the cell surface (see Figure 3). These results demonstrated that the parasite nucleosomal component represents the DC-stimulatory activity of malaria MZs.

### TLR9-mediates the activation of DCs by *P. falciparum* polynucleosomes and complex formation with histones is sufficient for the efficient activation of DCs by parasite DNA

**Figure 5:**
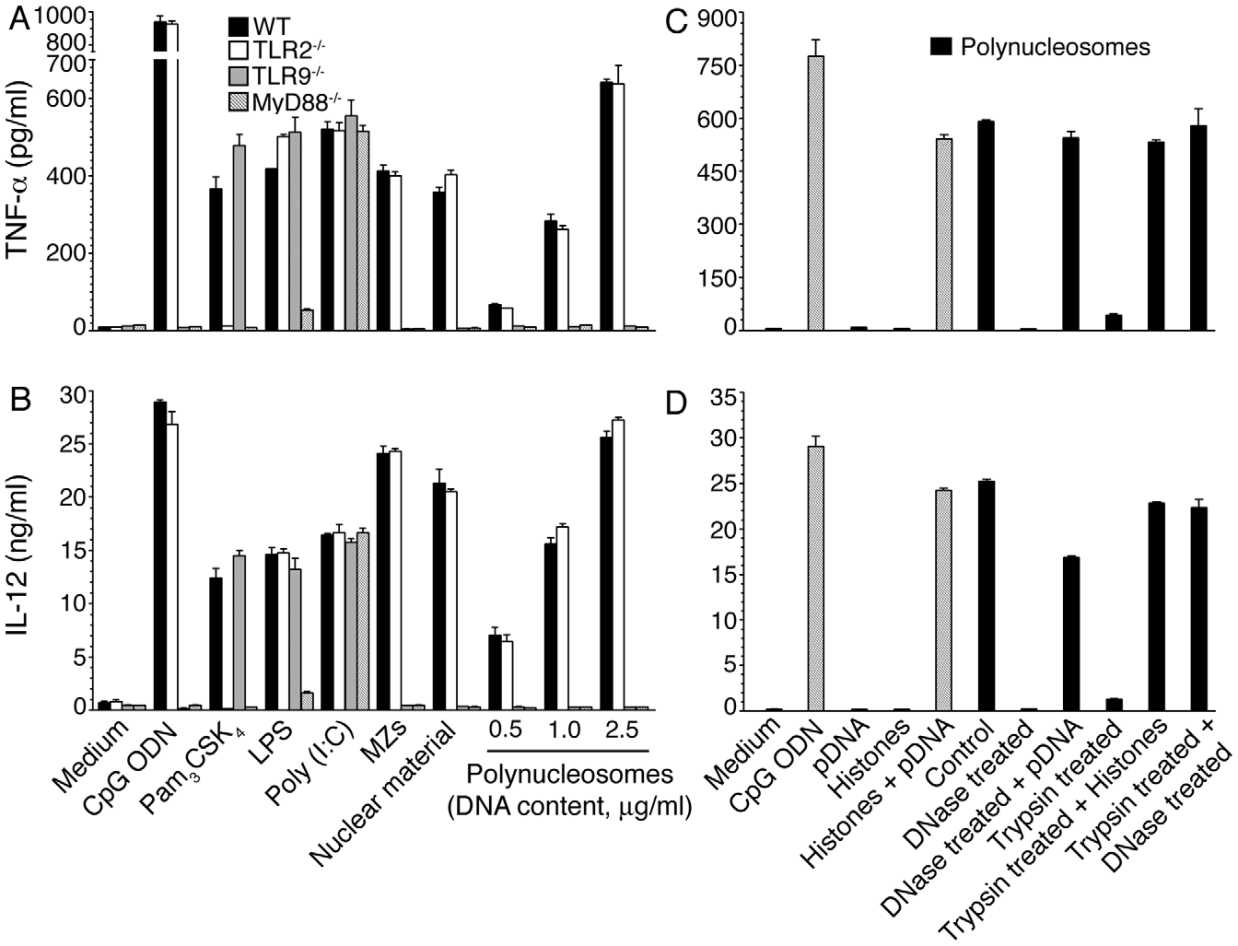
Analysis of the immunostimulatory activity and TLR specificity of *P. falciparum* polynucleosomes. **Panels A** and **B**: FL-DCs prepared from the bone marrow of WT, TLR2^−/−^, TLR9^−/−^ and MyD88^−/−^ mice were stimulated with the parasite nuclear material or with the indicated doses (based on DNA content) of polynucleosomes. FL-DCs stimulated with Pam_3_CSK_4_, Poly I∶C, LPS or CpG ODN were used as controls. The levels of TNF-α and IL-12 in the culture supernatants were measured by ELISA. **Panels C** and **D**: TNF-α and IL-12 produced by FL-DCs from WT mice stimulated with polynucleosomes, DNase-treated polynucleosomes, DNase-treated polynucleosomes to which parasite genomic DNA (pDNA) was added, trypsin-treated polynucleosomes, trypsin-treated polynucleosomes to which histones (1 µg/ml) were added or mixture of DNase- and trypsin-treated polynucleosomes. The culture supernatants of DCs stimulated with 8 µg/ml parasite genomic DNA (pDNA) and CpG ODN were analyzed as controls. Experiments were repeated three times and each time performed in duplicates. Error bars represent mean values ± SEM.

**Figure 6:**
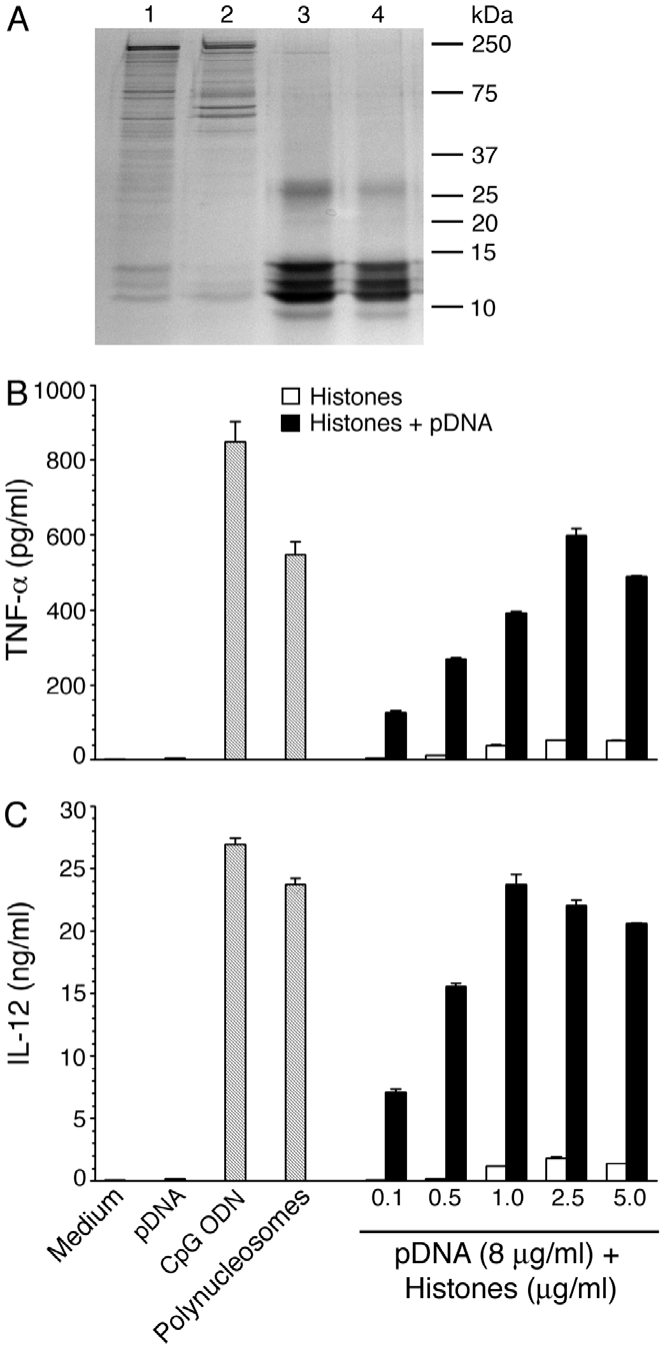
*P. falciparu*m histone-DNA complex efficiently activate DCs. **Panels A**: The parasite chromatin material, 0.8 M NaCl extract of chromatin material, and 0.25 M HCl extracts of chromatin material (Figure 1) were analyzed by SDS-PAGE using 15% gels. Each lane was loaded with 10 µg of protein. Lane 1, polynucleosomes; lane 2, 0.8 M NaCl extract of the chromatin material; lane 3 and 4, 0.25 M HCl extracts (histones). The mobility of molecular weights of marker proteins is indicated to the right. **Panels B** and **C**: TNF-α and IL-12 produced by FL-DCs obtained from WT mice stimulated with different doses of isolated parasite histones with or without parasite genomic DNA (pDNA, 8 µg/ml). DCs stimulated with parasite genomic DNA (pDNA, 8 µg/ml), CpG (2 µg/ml) and polynucleosomes (2.5 µg DNA content/ml) were analyzed as controls. Error bars represents mean values ± SEM.

TLR9 has been shown to be the specific receptor for the recognition of microbial DNA [45], [46]. To determine the receptor recognition specificity of polynucleosomes, we tested their stimulatory activity using FL-DCs prepared from the bone marrows of TLR9^−/−^ and MyD88^−/−^ mice; DCs from WT and TLR2^−/−^ mice were used as controls. Polynucleosomes efficiently activated the FL-DCs derived from WT and TLR2^−/−^ mice, producing similar levels of TNF-α and IL-12 (Figures 5A and 5B). In contrast, TLR9^−/−^ and MyD88^−/−^ DCs showed little or no activity, indicating that the activity of polynucleosomes is due to the TLR9-specific recognition of DNA. To further demonstrate that DNA is the active molecule, polynucleosomes were treated with DNase. This treatment resulted in the complete loss of DC-stimulatory activity of polynucleosomes (Figures 5C and 5D). The addition of the purified parasite genomic DNA to the DNase-treated polynucleosomes fully restored the activity, indicating that DNA is the active component. Treatment with trypsin also abolished almost all of the DC-stimulatory activity of polynucleosomes (Figures 5C and 5D). The addition of histones to the trypsin-treated polynucleosomes completely restored the activity. Further, the activity was also fully restored when DNase-treated and trypsin-treated polynucleosomes were combined together (Figures 5C and 5D). Studies have shown that DCs cannot internalize the highly negatively charged DNA molecules having extended structures [26], [47], [48]. However, complex formation with highly positively charged polypeptides leads to the formation of condensed structure, which is internalized by DCs, thereby presenting DNA to TLR9 in the endosomal compartment [48]. Consistent with these observations, while parasite genomic DNA alone was unable to activate DCs, the addition of exogenous (calf thymus) histones conferred activity to the genomic DNA (Figure S2). To conclusively demonstrate that histone-DNA complex is the actual active component, we purified parasite histones by extracting the nuclear material with 0.8 M NaCl to remove most of the loosely associated proteins (Figure 6A, lane 2) followed by 0.25 M HCl extraction. Other parasite proteins were either absent or present at very low levels in the histone preparations (Figure 6A, lanes 3 and 4); the protein band at ∼27 kDa has similar molecular weight corresponds to H1 histone, whereas those below 15 kDa are H3, H2A, H2B and H4, respectively. The mixture of purified parasite histones (Figure 6A) and genomic DNA free of parasite proteins efficiently activated DCs to produce inflammatory cytokines (Figures 6B and 6C). Thus, collectively, these data demonstrated that parasite histone-DNA complex is the TLR9-dependent DC-activating constituent of malaria parasite nuclear material.

**Figure 7:**
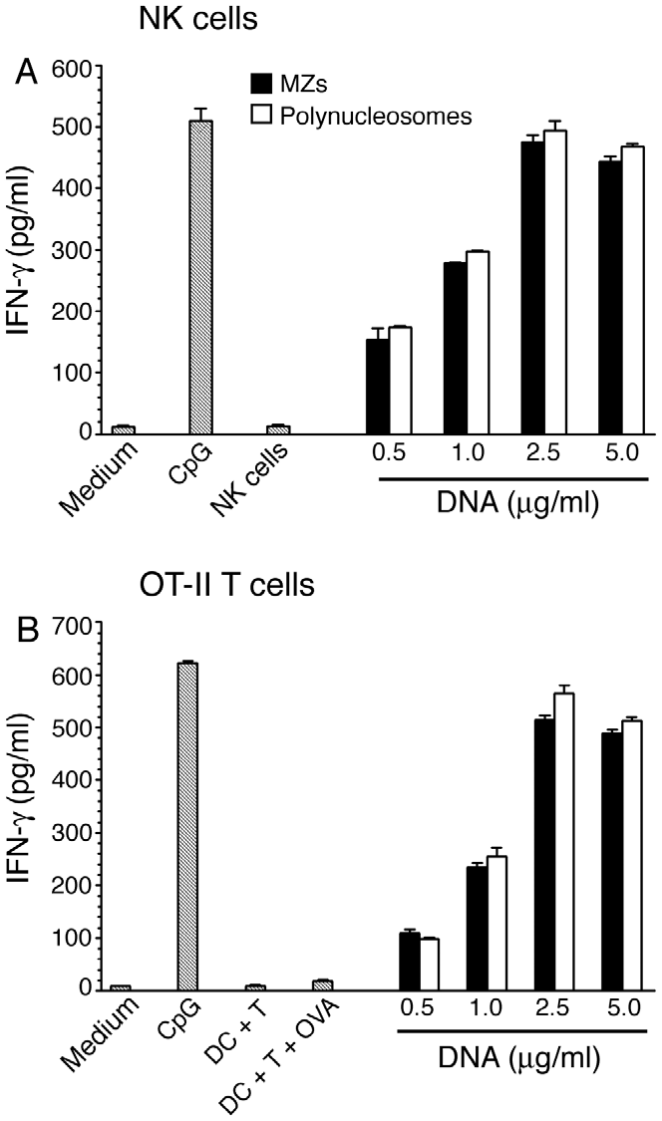
*P. falciparu*m polynucleosomes-activated DCs stimulate NK cells and OT-II T cells to produce IFN-γ. **Panel A**: WT FL-DCs were cocultured with NK cells and stimulated with the indicated doses of polynucleosomes (based on DNA content). After 36 h, the IFN-γ produced by NK cells was measured by ELISA. Merozoites (MZs) having the indicated DNA contents and CpG ODN were used as controls. **Panel B**: WT FL-DCs were stimulated with the indicated doses of polynucleosomes for 6 h and then cocultured with OT-II T cells in the presence of OVA^323–339^ peptide. After 72 h, the IFN-γ produced by T cells was measured by ELISA. In parallel NK cells, DCs or T cells were cultured individually or cocultures of DCs and T cells in presence or absence of OVA^323–339^ peptide, but not stimulated with polynucleosomes, were used as controls. Data are representative of two independent experiments, each performed in duplicates. Error bars represents mean values ± SEM.

Previously, we showed that DCs stimulated with *P. falciparum* MZs could activate NK cells to induce the production of IFN-γ [26]. To determine whether this activity of MZs is also localized to polynucleosomes, we analyzed IFN-γ production by NK cells after stimulation with DCs activated by polynucleosomes. The NK cells efficiently produced IFN-γ (Figure 7A). On the DNA equivalent basis, the levels of IFN-γ produced by NK cells stimulated with polynucleosome-treated DCs were comparable with those produced by MZ-treated coculture of DCs and NK cells. Similarly, the polynucleosome-stimulated DCs, primed with OVA^323–339^ peptide, could activate OT-II T cells to produce IFN-γ (Figure 7B); the IFN-γ levels produced were comparable to those by OT-II T cells cocultured with MZ-stimulated and OVA^323–339^ peptide-primed DCs. Thus, these results indicate that DC-stimulating activity of MZs is localized to polynucleosomes.

### Mononucleosomes and oligonucleosomes of *P. falciparum* efficiently activate DCs

**Figure 8:**
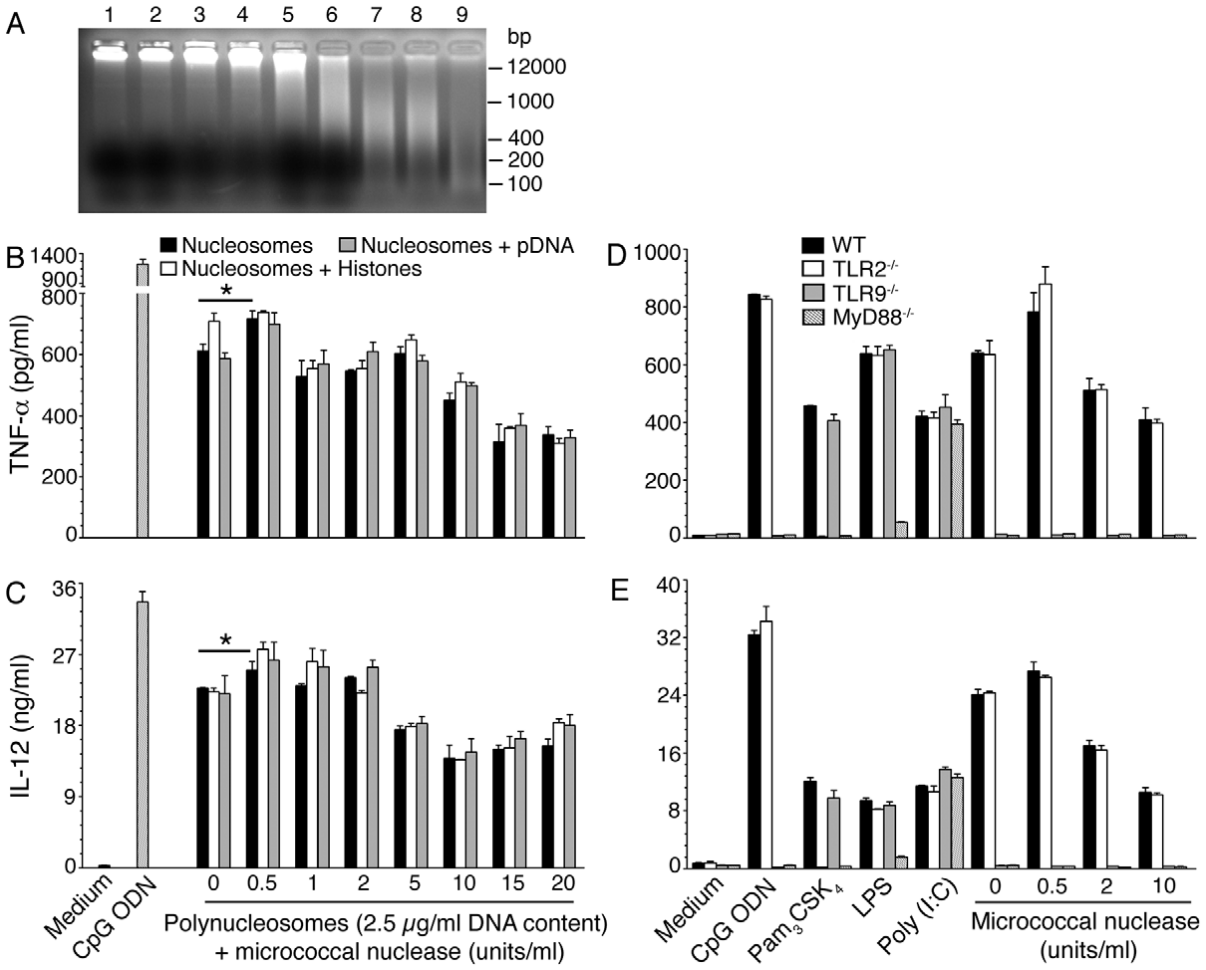
The *P. falciparum* mononucleosomes and oligonucleosomes efficiently activate DCs to induce the production of proinflammatory cytokines. **Panel A**: The parasite polynucleosomes (15 µg DNA content in 200 µl buffer) were treated with the indicated concentrations of micrococcal nuclease and 10 µl aliquots of the enzyme-digestion products (0.75 µg DNA content) were analyzed by electrophoresis using 2% agarose gels. Lane 1, untreated polynucleosomes; lane 2, polynucleosomes incubated with buffer only; lanes 3 to 9, respectively were polynucleosomes treated with 0.5, 1.0, 2.0, 5.0, 10, 20 and 50 units/ml of micrococcal nuclease. The sizes of DNA standards are indicated to the right. **Panels B** to **E**: TNF-α and IL-12 produced by WT FL-DCs stimulated with mono- or oligonucleosomes obtained by different doses of micrococcal nuclease treatment of polynucleosomes (**panels B** and **C**) or FL-DCs from WT, TLR2^−/−^, TLR9^−/−^ and MyD88^−/−^ stimulated with parasite oligonucleosomes (**panels D** and **E**). FL-DCs stimulated with Pam_3_CSK_4_, Poly I∶C, LPS or CpG ODN were analyzed as controls. The analysis was performed three times each time in duplicates, and results of a representative experiment are shown. Error bars represent mean values ± SEM. * *p*<0.05, comparison between 0.5 units/ml of micrococcal nuclease digested and undigested polynucleosomes.

To determine the length of polynucleosomes required for the DC-stimulatory activity, we prepared mononucleosomes and oligonucleosomes ranging in size from 2 to several mononucleosomes by treating polynucleosomes with various concentrations of micrococcal nuclease. Electrophoretic analysis of the enzyme digestion products revealed that the enzyme gradually converted polynucleosomes into oligonucleosomes, as indicated by the decrease in the amounts of high molecular size polynucleosomes that could barely enter gels, eventually forming mono and oligonucleosomes having 2–8 mononucleosomes (Figure 8A). Treatment with low concentrations of micrococcal nuclease (0.5 units/ml) caused a significant increase in the stimulatory activity of polynucleosomes (Figures 8B and 8C). This is presumably due to the decreased size of polynucleosomes, facilitating uptake by DCs and thus resulting in higher level of polynucleosomes entering and efficiently activating the cells. Treatment with increased concentrations of micrococcal nuclease resulted in the formation of mononucleosomes (DNA size ∼100 bp, lane 9 in Figure 8A) and oligonucleosomes (consists of 2–8 mononucleosomes, DNA size 300–1200 bp; lanes 7 and 8 in Figure 8A). On the basis of DNA content, the activity of mononucleosomes and oligonucleosomes was ∼70% of the stimulatory activity of polynucleosomes (Figures 8B and 8C) and the activity was TLR9 specific (Figures 8D and 8E). The addition of either histones or purified parasite genomic DNA to untreated polynucleosomes and micrococcal nuclease-treated polynucleosomes had no effect on their stimulatory activity (Figures 8B and 8C). These results suggested that polynucleosome preparations do not contain significant levels of histone-unbound DNA or free histones. The moderate loss of activity upon micrococcal nuclease treatment is likely due to the cleavage of the exposed linker DNA regions of polynucleosomes. The results suggested that the nucleotide sequences of the linker DNA regions of polynucleosomes also contain specific DNA motifs that are efficiently recognized by TLR9. Alternatively, the linker DNA regions of chromatin material are more efficiently recognized by TLR9 because they are not tightly complexed with histones as in the case of nucleosomes.

### Parasite protein-DNA complex represents the major TLR9-specific activity of *P. falciparum* merozoite nuclear material

**Figure 9:**
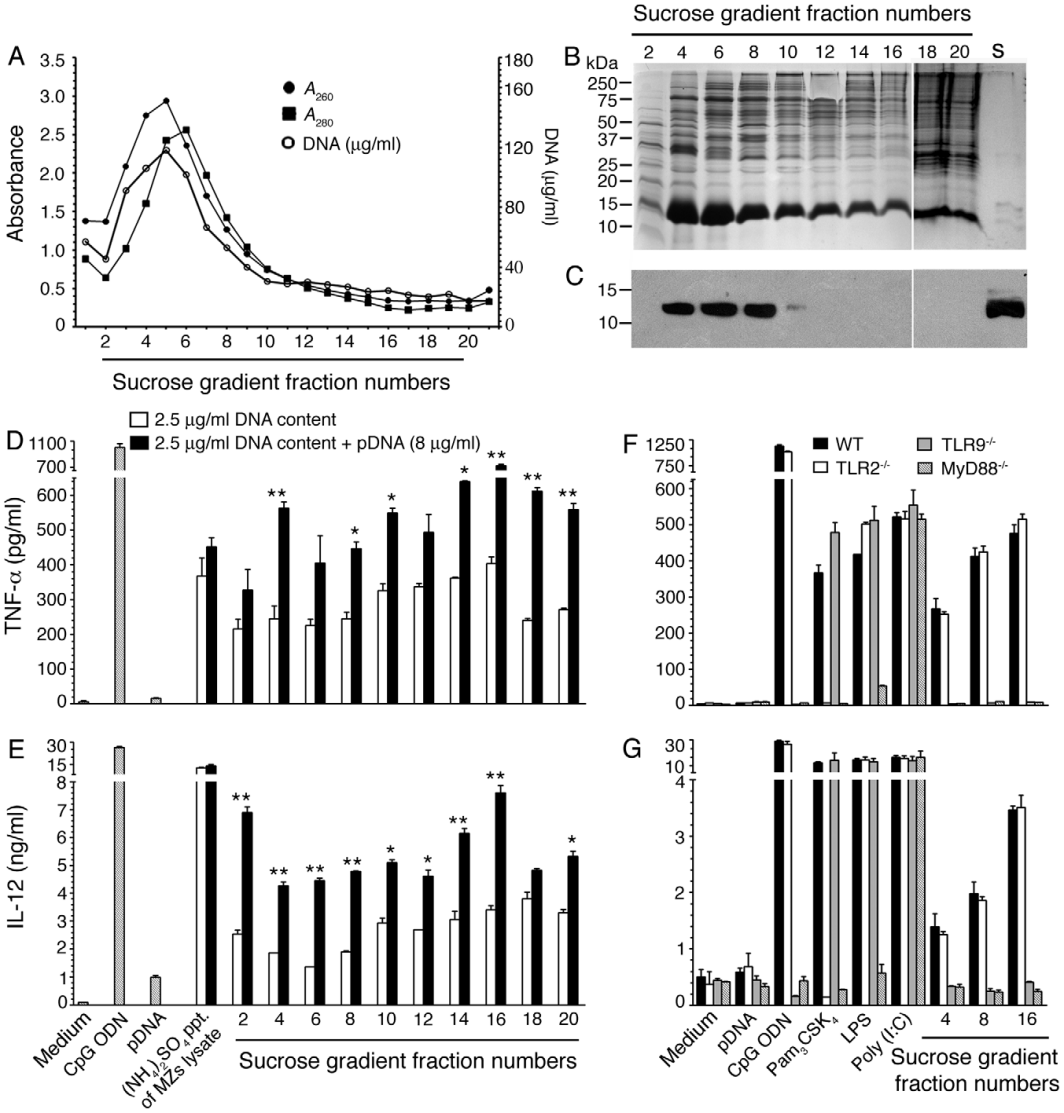
*P. falciparum* proteins other than histones confer stimulatory activity to the parasite DNA. **Panel A**: Fractionation of MZs lysate by sucrose-density gradient centrifugation. The values of absorption at 260 nm (ℓ) and 280 nm (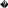), and DNA contents (O) of the fractions were measured as described in “Materials and Methods”, and plotted. **Panel B**: SDS-PAGE analysis. The sucrose gradient fractions were dialyzed; aliquots corresponding to 20 µg of proteins were analyzed by SDS-PAGE using 15% gels. Sucrose gradient fraction numbers are indicated at the top. The mobility of molecular weight marker proteins is indicated to the left. Lane S, a mixture of standard H1, H2A, H2B, H3 and H4 histones. **Panel C**: Western blotting of sucrose gradient fractions using anti-H3 histone polyclonal antibodies. Lane descriptions are same as that in **panel B**. **Panels D** and **E**: TNF-α and IL-12 produced by WT FL-DCs stimulated with the indicated sucrose gradient fractions having 2.5 µg/ml of DNA content or with fractions having 2.5 µg/ml of DNA content to which the purified parasite genomic DNA (pDNA, 8 µg/ml) was added. Cells stimulated with parasite genomic DNA were used as controls. **Panels F** and **G**: TNF-α and IL-12 produced by FL-DCs from WT, TLR2^−/−^, TLR9^−/−^ and MyD88^−/−^ stimulated with sucrose gradient fractions. DCs stimulated with Pam_3_CSK_4_, Poly I∶C, LPS, CpG ODN or parasite genomic DNA were used as a control. The analysis was repeated three times each time performed in duplicates, and results of a representative are shown. Error bars represent mean values ± SEM. * *p*<0.05; ** *p*<0.01 comparison between sucrose gradient fractions with and without added parasite DNA.

To further ascertain that the TLR9-specific DC stimulatory activity of MZs corresponds to the nuclear protein-DNA complex, we lysed MZs by freezing and thawing, and the lysate was fractionated by sucrose-density gradient centrifugation. Measurement of UV absorption at 260 and 280 nm in various fractions showed that most of the proteins and DNA are concentrated at the top one-third portion of the sucrose gradient, while the lower two-third of the gradient contained low levels of proteins and DNA (Figure 9A). Agarose gel electrophoresis showed that DNA is present mainly in fractions 4–10 (Figure S3). SDS-PAGE analysis revealed that proteins of various sizes are present in all fractions of the sucrose gradient. Although, proteins having the sizes similar to histones were observed in all fractions, Western blotting using anti-H3 histone antibodies indicated the presence of histones only in fractions corresponding to peak levels of DNA but not in denser fractions containing low levels of DNA (Figures 9B and 9C). Upon activity analysis, in additions to fractions (4–10 in Figure 9A) having high levels of histones and DNA, those fractions (12–20 in Figure 9A) in which histones are either absent or present at low levels also activated DCs to produce TNF-α and IL-12 (Figures 9D and 9E). The addition of parasite genomic DNA, significantly increased the stimulatory activity of all fractions, producing high levels of TNF-α and IL-12. The activity of these fractions was TLR9-dependent (Figures 9F and 9G) as DCs deficient in TLR9 unable to produce cytokines. Further, treatment with DNase abolished >95% of the activity of the fractions in WT DCs and addition of parasite genomic DNA to the enzyme-treated fractions restored the TLR9-dependent activity (Figure S4). Since histones were either absent or present at low levels in fractions 12–20, the observed DC-stimulatory activity of these fractions upon the addition of genomic DNA could be due to complexing of DNA with parasite proteins other than histones. However, the possibility of histones being present at low levels that efficiently complex with DNA, conferring the activity cannot be ruled out. Nevertheless, overall, these results agree with the results shown in Figures 2, 6 and 8, confirming that nuclear material containing histone-DNA complex is the major DC-activating component of *P. falciparum* MZs.

## Discussion

The components of malaria parasite that activate DCs through the recognition of TLR9 have been controversial [26], [47], [49], [50]. Earlier studies have shown that either hemozoin alone or hemozoin-DNA could activate DCs in a TLR9-dependent manner [47], [50]. However, we recently demonstrated that hemozoin is an inert material and it neither activates DCs through TLR9-mediated signaling nor functions as a carrier of DNA into cells [26]. We have also demonstrated that MZs are the major factor among the components released during the *P. falciparum* schizont burst that activate DCs *via* TLR9. Furthermore, we have showed that protein-DNA complex is the major DC-activating constituent of MZs. In the present study, we obtained multiple evidence that allowed us to conclude that the histone-DNA complex of the parasite chromatin material constitutes the TLR9-mediated immunostimulatory activity of MZs that triggers the activation of DCs leading to the downstream production of inflammatory cytokines. The evidence in support of our conclusion includes: (i) Similar to the whole MZs, the parasite nuclear material or polynucleosomes possess a robust immunostimulatory activity to stimulate DCs. The ability of the parasite nuclear material or polynucleosomes to induce the production of proinflammatory cytokines, TNF-α and IL-12 by DCs, was comparable to that of MZs. (ii) Like MZs, the nuclear material or polynucleosomes efficiently upregulated the surface expression of costimulatory molecules such as CD40, CD80 and CD86 in DCs. (iii) The mononucleosomes and oligonucleosomes prepared by the digestion of parasite polynucleosomes with micrococcal nuclease could efficiently activate DCs to produce proinflammatory cytokines. (iv) The digestion of polynucleosomes with DNase resulted in the complete loss of stimulatory activity and the activity was fully restored upon the addition of parasite genomic DNA to the enzyme-treated samples (see Figure 5). (v) The treatment of polynucleosomes with trypsin also abolished the stimulatory activity and the activity was completely regained when exogenous histones were added to the trypsin-treated samples. (vi) The mixture of the DNase-digested polynucleosomes and the trypsin-treated polynucleosomes could efficiently activate DCs; on the basis of DNA content, the stimulatory activity of the mixture was comparable to that of untreated polynucleosomes (see Figure 5). (vii) Finally, the purified parasite genomic DNA, which cannot by itself enter and activate DCs [26], showed efficient stimulatory activity upon the addition of purified parasite histones. These data demonstrate that histone-DNA complex of parasite nucleosomes is the TLR9-specific, DC-activating factor of malaria parasites.

Previously, other protozoan parasites such as *Toxoplasma gondii*, *Trypanosoma cruzii* and *Leishmania major* have been shown to activate DCs through the DNA-mediated recognition of TLR9 [51]–[56]. However, how the negatively charged DNA with a highly extended structure gain entry into cells has not been reported. The results of this study clearly show that complexing of DNA with proteins, presumably to form condensed DNA structures, is essential for the facile entry of DNA to the endosomal compartments. Our findings agree with the results of a previous study that, in bacteria-induced skin psoriasis, DNA complexes with the bacterial cationic amphipathic peptide, LL37 and forms a condensed DNA structure, thereby efficiently entering into DCs and activating cells through TLR9-mediated signaling and producing inflammatory cytokines [48]. In fact, the property of LL37 and other α-helical cationic peptide to form a tight complex with DNA has been exploited for the transfection of DNA to cells [57]–[60].

In addition to the histone-DNA complex, nonhistone proteins of *P. falciparum* appear to complex with DNA, thereby activating DCs in a TLR9-dependent manner to induce the production of proinflammatory cytokine responses. This is obvious by our observations that buffer/salt extracts of parasite nuclear material that contain little or no histone exhibit activity when supplemented with the purified parasite genomic DNA (see Figure S1). In addition, the fractions from the sucrose density gradient centrifugation of parasite lysates that contain DNA but either lack or have very low levels of histones could stimulate DCs to produce inflammatory cytokines (see Figure 9). MZs released by the burst of schizont stage malaria parasites are labile. Unless they quickly invade erythrocytes or immediately taken up by phagocytosis, MZs probably undergo lysis *in vivo* [36]–[43]. Since malaria parasites are transcriptionally active throughout their blood stage development, it is likely that, upon parasite lysis, euchromatin region containing DNA as an extended structure not complexed with histones is released by shearing. The positively charged nonhistone parasite proteins can complex with these free DNA segments, forming protein-DNA complex, which activates DCs in a manner similar to histone-DNA complex of nucleosomes. Although our results point out that parasite proteins other than histones can also confer activity to DNA, their contributions as compared to histones may be minor.

In conclusion, the results of the present study demonstrate that histone-DNA complex of the nucleosome is the major TLR9-dependent immunostimulatory, DC-activating component of *P. falciparum*. The polynucleosomes as well as mono- and low molecular weight oligonucleosomes can efficiently activate DCs in a TLR9-specific manner to induce the production of proinflammatory cytokines. Our results also suggest that, to certain extent, parasite proteins other than histones, those presumably having net positive charges, may form complex with parasite DNA, activating DCs to produce cytokines. Overall, our results have important implications for the development of immunotherapeutics and/or an effective vaccine for malaria.

## Supporting Information

Figure S1 **Non-histone proteins of** ***P. falciparum*** **confer low level of stimulatory activity to parasite genomic DNA.** **Panels A to D**: TNF-α and IL-12 produced by WT FL-DCs stimulated with the parasite cytoplasmic material plus membrane fragments or buffer/salt extracts (see **Figure 1**) with or without added parasite genomic DNA (pDNA). DCs stimulated with MZs lysate nuclear material (see **Figure 1**, 2.5 µg/ml DNA content) or CpG ODN (2 µg/ml) was used as controls. Data are representative of two independent experiments, each performed in duplicates. Error bars represent mean values ± SEM.
(TIF)

Figure S2 **Histones are the major carriers of** ***P. falciparum*** **DNA entry into DCs to induce cytokine responses.** **Panels A** and **B**: TNF-α and IL-12 produced by WT FL-DCs stimulated with parasite genomic DNA (pDNA) plus the indicated doses of histones were measured using ELISA. Cells similarly stimulated with CpG ODN were used as a control. Data are representative of two independent experiments and each time done in duplicates.
(TIF)

Figure S3 **Agarose gel electrophoresis of sucrose gradient fractions.** The sucrose gradient fractions (see Figure 9) were analyzed for DNA by 1% agarose gel electrophoresis. The fraction numbers are indicated at the top of the panel. The mobility of DNA molecular marker standards is indicated to the right.
(TIF)

Figure S4 **The stimulatory activity in the sucrose gradient fractions is due to DNA.** **Panels A** and **B**: TNF-α and IL-12 produced by WT FL-DCs stimulated with sucrose density gradient fractions (see Figure 9; 2.5 µg/ml DNA content). The sucrose gradient fractions were also analyzed after treatment with DNase with or without the addition of purified genomic DNA (pDNA, 8.0 µg/ml). The culture supernatants of DCs stimulated with 8.0 µg/ml of genomic DNA (pDNA) alone or CpG ODN (2 µg/ml) were used as controls. Data are representative of two independent experiments, each performed in duplicates. Error bars represent mean values ± SEM.
(TIF)
