# Supplementary figures and images for: The Nucleosome (Histone-DNA Complex) Is the TLR9-Specific Immunostimulatory Component of Plasmodium falciparum That Activates DCs
Source: PLoS One. 2011 Jun 8;6(6):e20398. doi: 10.1371/journal.pone.0020398 (PMC3110622; doi:10.1371/journal.pone.0020398)

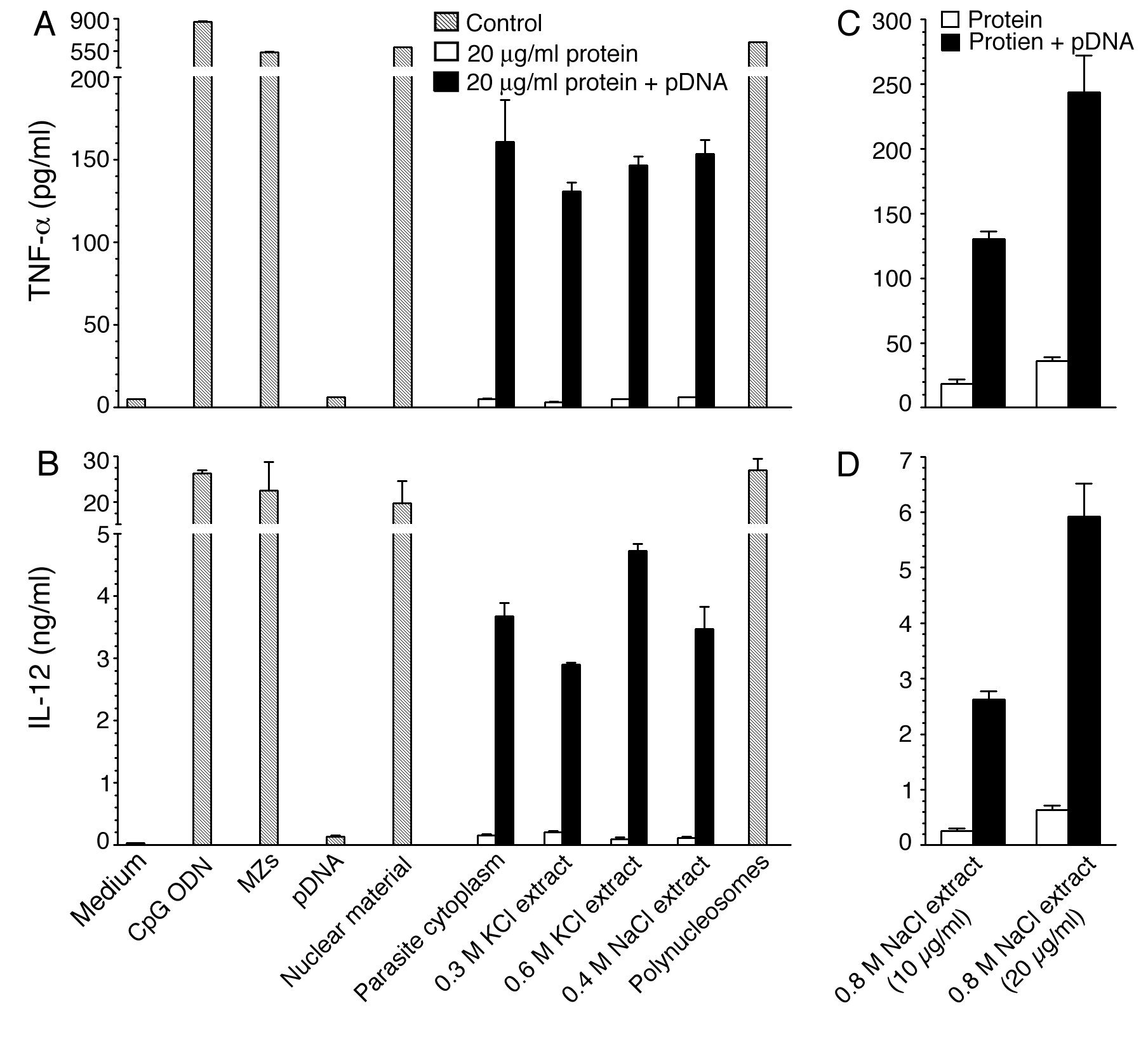

Supplement: Figure S1 — Non-histone proteins of P. falciparum confer low level of stimulatory activity to parasite genomic DNA. Panels A to D: TNF-α and IL-12 produced by WT FL-DCs stimulated with the parasite cytoplasmic material plus membrane fragments or buffer/salt extracts (see Figure 1 ) with or without added parasite genomic DNA (pDNA). DCs stimulated with MZs lysate nuclear material (see Figure 1 , 2.5 µg/ml DNA content) or CpG ODN (2 µg/ml) was used as controls. Data are representative of two independent experiments, each performed in duplicates. Error bars represent mean values ± SEM. (TIF) [file pone.0020398.s001.tif]

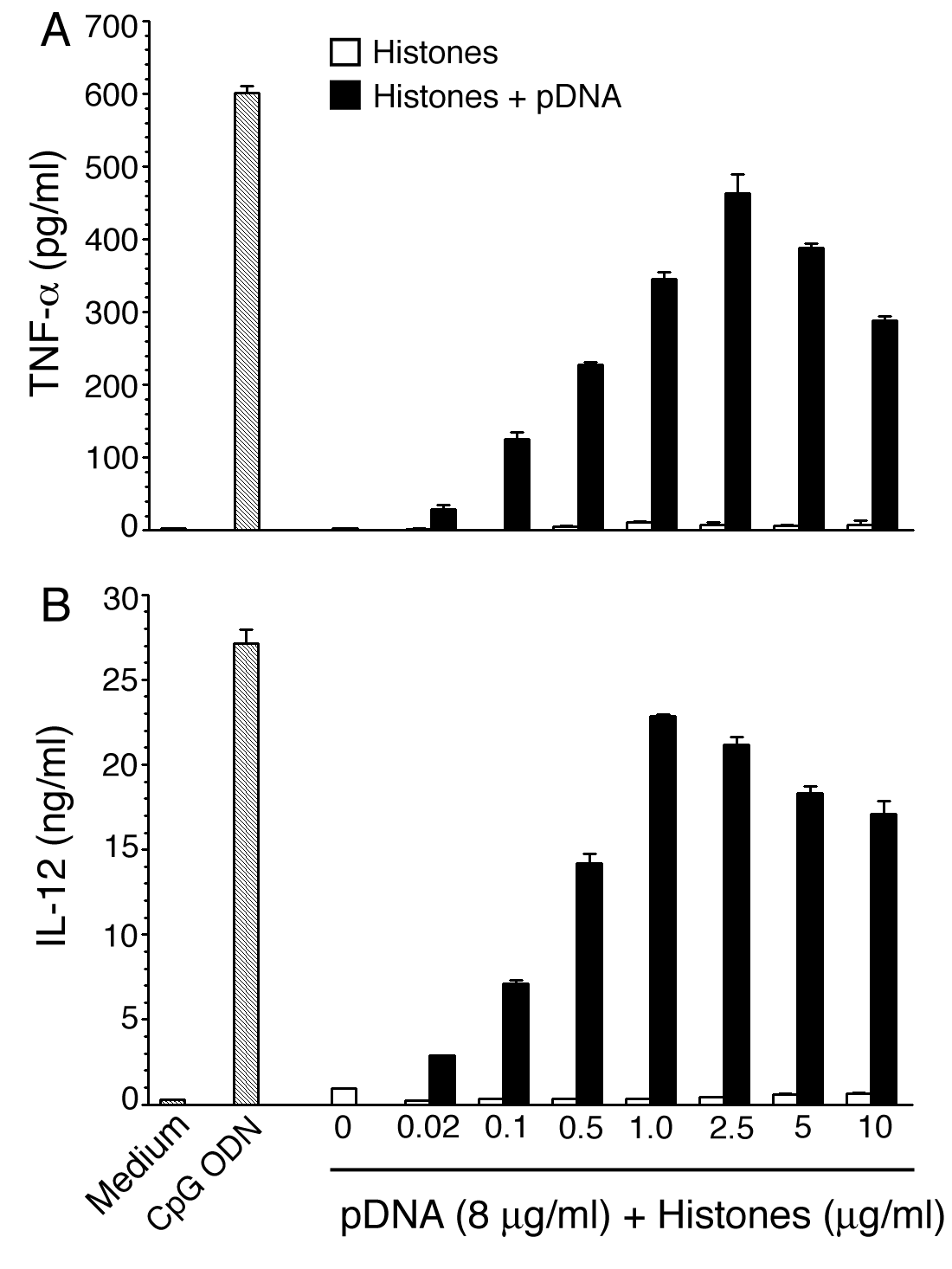

Supplement: Figure S2 — Histones are the major carriers of P. falciparum DNA entry into DCs to induce cytokine responses. Panels A and B: TNF-α and IL-12 produced by WT FL-DCs stimulated with parasite genomic DNA (pDNA) plus the indicated doses of histones were measured using ELISA. Cells similarly stimulated with CpG ODN were used as a control. Data are representative of two independent experiments and each time done in duplicates. (TIF) [file pone.0020398.s002.tif]

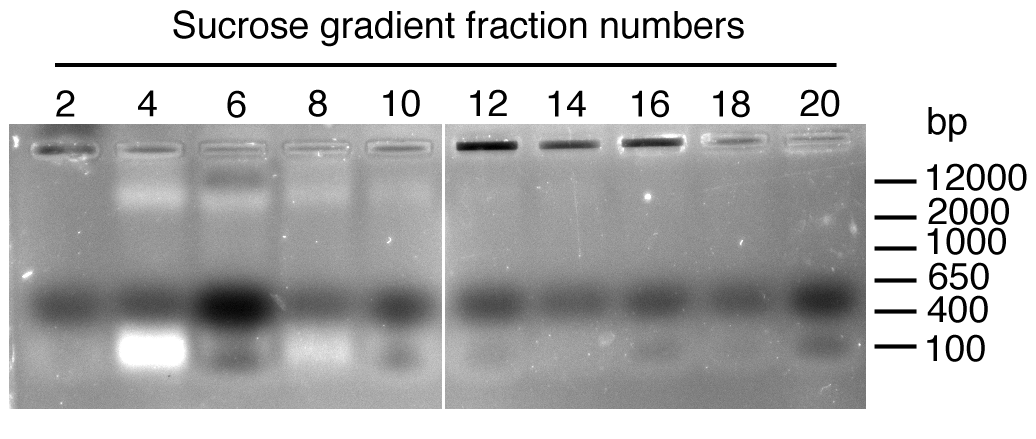

Supplement: Figure S3 — Agarose gel electrophoresis of sucrose gradient fractions. The sucrose gradient fractions (see Figure 9) were analyzed for DNA by 1% agarose gel electrophoresis. The fraction numbers are indicated at the top of the panel. The mobility of DNA molecular marker standards is indicated to the right. (TIF) [file pone.0020398.s003.tif]

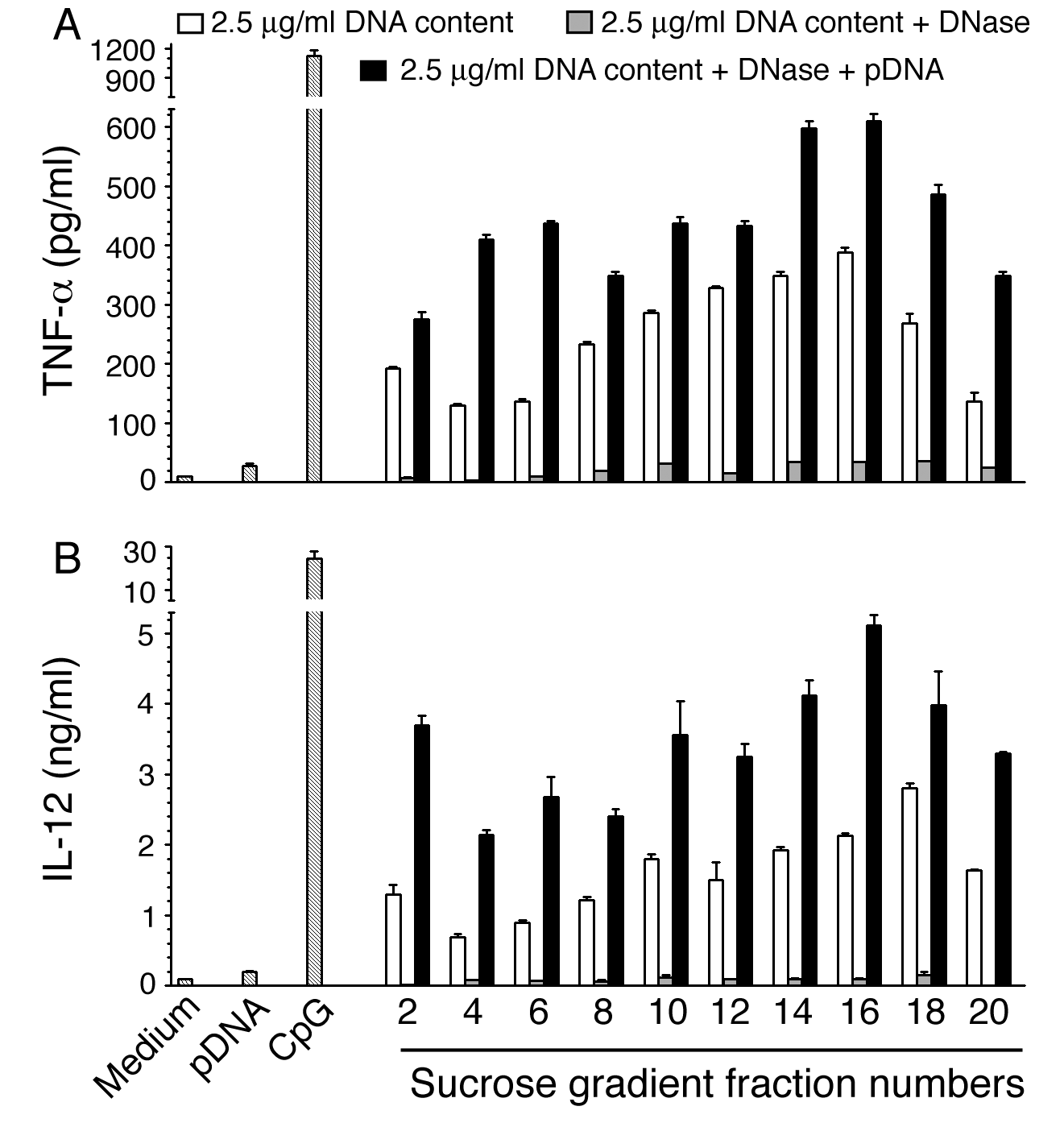

Supplement: Figure S4 — The stimulatory activity in the sucrose gradient fractions is due to DNA. Panels A and B: TNF-α and IL-12 produced by WT FL-DCs stimulated with sucrose density gradient fractions (see Figure 9; 2.5 µg/ml DNA content). The sucrose gradient fractions were also analyzed after treatment with DNase with or without the addition of purified genomic DNA (pDNA, 8.0 µg/ml). The culture supernatants of DCs stimulated with 8.0 µg/ml of genomic DNA (pDNA) alone or CpG ODN (2 µg/ml) were used as controls. Data are representative of two independent experiments, each performed in duplicates. Error bars represent mean values ± SEM. (TIF) [file pone.0020398.s004.tif]
